# The Demonstration of an Aqp4/Tgf-Beta 1 Pathway in Murine Astrocytes Holds Implications for Both Neuromyelitis Optica and Progressive Multiple Sclerosis

**DOI:** 10.3390/ijms21031035

**Published:** 2020-02-04

**Authors:** Serge Nataf

**Affiliations:** 1 Bank of Tissues and Cells, Lyon University Hospital (Hospices Civils de Lyon), F-69000 Lyon, France; serge.nataf@inserm.fr; Tel.: +33-4-72-11-76-67; Fax: 33-4-72-11-96-49; 2 CarMeN Laboratory, INSERM 1060, INRA 1397, 69600 INSA Oullins, France; 3 Lyon-Est School of Medicine, University Claude Bernard Lyon-1, F-69000 Lyon, France

**Keywords:** aquaporin-4, TGF-beta 1, neuromyelitis optica: multiple sclerosis, angiotensin II

## Abstract

The role exerted by Aquaporin 4 (AQP4) as a regulator of astrocyte immune functions has been poorly explored. A recent report demonstrates that under neuroinflammatory conditions, the expression of Aqp4 on murine astrocytes is mandatory for the effective control of acute inflammation in the central nervous system. Such an immunomodulatory function appears to be mediated by a promotion of the transforming growth factor beta 1 (Tgfb1) pathway. Here, these results are discussed in the context of neuromyelitis optica (NMO) and multiple sclerosis (MS) progressive forms. It is proposed that NMO and progressive MS might rely on opposite molecular mechanisms involving, in NMO, an acutely-defective AQP4/TGFB1 pathway and, in progressive MS, a chronically-stimulated AQP4/TGFB1 pathway. Data supporting the involvement of angiotensin II as a molecular link between AQP4 and TGFB1 are also reviewed.

In a recent paper, Xue et al. established that under conditions of acute neuronal insult, mice knock out for aquaporin 4 (*Aqp4*) exhibit an impaired systemic and intra-cerebral synthesis of transforming growth factor-beta 1 (Tgfb1) [1]. As a consequence, the failed Tgfb1-mediated dampening of microglial activation is responsible for an increase of both inflammation and neuronal alterations [1]. This mini-review/commentary is primarily intended to discuss these results in the context of two neuroinflammatory conditions affecting the spinal cord: multiple sclerosis and the anti-AQP4 autoantibody-mediated disorder neuromyelitis optica (NMO) [2]. The paper by Xue et al. not only brings an additional experimental proof that Tgfb1 is a potent inhibitor of acute CNS inflammation [3,4] but provides the first demonstration of an Aqp4/Tgf-β1 pathway in murine astrocytes. Extrapolating this finding to human astrocytes implies that anti-AQP4 autoantibodies in NMO patients might drive a failure of the AQP4/TGFB1 anti-inflammatory axis. In turn, this would lead to an uncontained inflammatory reaction. Favoring this view, it is important to remind that NMO is characterized by the development of fast-evolving sub-acute spinal cord lesions which frequently cover large areas on the longitudinal axis [5] and exhibit neuropathological signs of pronounced inflammation [6]. While anti-AQP4 autoantibodies in NMO patients undoubtedly exert cytotoxic effects on astrocytes [7,8], other pathophysiological mechanisms relying on an alteration of AQP4 functions, have been incriminated [9,10]. Since several non-cytotoxic effects have been firmly demonstrated in astrocytes exposed to anti-AQP4 antibodies, it would be interesting to determine whether such autoantibodies may also impact the ability of cultured human astrocytes to synthesize TGFB1. Besides NMO, the demonstration of an AQP4/TGFB1 axis may also provide new clues regarding the pathophysiology of MS progressive forms. In the spinal cord of patients suffering from primary progressive or secondary progressive MS, we demonstrated the existence of large areas of periplaque astrocytosis, which extend distance away from plaque border. Such areas of tissue remodeling are characterized by an up-regulation of AQP4 [11] and a progliotic TGFB1 molecular signature [12,13,14]. We proposed that the chronic overexpression of TGFB1, while efficiently containing acute inflammation in MS spinal cords, may promote astrocytosis, partial demyelination and low grade chronic inflammation via at least two main mechanisms: (i) the astrocytic synthesis of profibrotic extracellular matrix proteins [15] and (ii) a direct inhibitory effect of TGFB1 on both myelin synthesis [13] and oligodendrogenesis [16]. The demonstration of an Aqp4/TgfB1 pathway in murine astrocytes reinforces this view and further suggests that any CNS condition characterized by an up-regulation of AQP4 on astrocytes might similarly involve the AQP4/TGFB1 pathway. Experimental evidence indicates this could be the case for the chronic gliotic processes observed in patients suffering from temporal lobe epilepsy [17,18] or developing a post-traumatic glial scar [19,20]. In any case, the fact that spinal cord appears to be preferentially targeted by gliosis in MS progressive forms deserves particular attention. Indeed, previous neuropathological studies indicated that spinal cord lesions in MS progressive forms tend to be less inflammatory than their brain counterparts [21,22]. While TGFB1-mediated inhibition of acute inflammation in the spinal cord might be favored by the regionalized expression of the homeobox gene HOXA5 [14], an additional explanation, not exclusive from the former, could be that spinal cord astrocytes might be more prone to an engagement of the AQP4/TGFB1 pathway, as compared to brain astrocytes. In this regard, it should be kept in mind that, although AQP4 is expressed on astrocytes throughout the central nervous system (CNS), AQP4-expressing spinal cord astrocytes are by far the main targets of anti-AQP4 antibodies in NMO patients. While not fully understood, such a peculiar distribution of CNS lesions was proposed to essentially rely on region-specific differences regarding the level of expression and the sub-cellular localization of AQP4 on astrocytes [23,24]. It is thus thinkable that spinal cord-specific molecular features shape the AQP4/TGFB1 pathway in a region-specific manner. Finally, further assessing the molecular links between AQP4 and TGFB1 appears essential. Interestingly, a quick data mining search using the gene co-expression analysis tool Multi Experiment Matrix (MEM) [25] shows that, across 2811 human microarray datasets, the list of 500 genes which most closely co-express with *AQP4* does not comprise *TGFB1*. The same result is obtained when exploring murine microarray datasets (n = 2401). These data mining findings suggest that *APQ4* may not directly impact the transcription of *TGFB1*. On another hand, according still to the MEM database and webtool, AQP4 tightly co-expresses with angiotensinogen (*AGT*) (*p*-value: 2.95^−41^, Pearson correlation test), a gene upregulated in MS spinal cords [11] and extensively shown to promote the TGFB1 pathway [26,27,28,29]. Similarly in mice, *Aqp4* tightly co-expresses with *Agt* (*p*-value: 5.85^−21^, Pearson correlation test). Of note, Angiotensin II, the main active metabolite of angiotensinogen, not only stimulates the transcription of TGFB1 [26,29] but exerts short-term activating effects on SMADs [30,31,32], the transducing molecules of the TGF-beta pathway. Along this line, under conditions of acute neuronal insult, the engagement of the angiotensin II receptor type 1 on murine astrocytes is mandatory to contain the influx of blood leucocytes through the blood brain barrier [33]. Finally, in the context of progressive MS, inhibitors of the angiotensin converting enzyme were proposed to be of therapeutic utility as off the shelf commercially-available TGFB1 inhibitors [12,13,14,16]. Altogether, these findings clearly urge to assess the molecular links between AQP4, AGT and TGFB1 in human astrocytes.

Overall, although NMO and progressive MS both stem from CNS-targeting autoimmune events, the evolution of spinal cord lesions in each disease appears to follow strikingly opposite fates: uncontained inflammation and rapid extension of lesions in NMO vs contained inflammation and slowly-expanding astrocytosis in MS. It is proposed here that NMO and progressive MS might rely on the following opposite molecular mechanisms: an acutely-defective AQP4/TGFB1 pathway in NMO versus a chronically-stimulated AQP4/TGFB1 pathway in progressive MS (Figure 1).

## Figures and Tables

**Figure 1:**
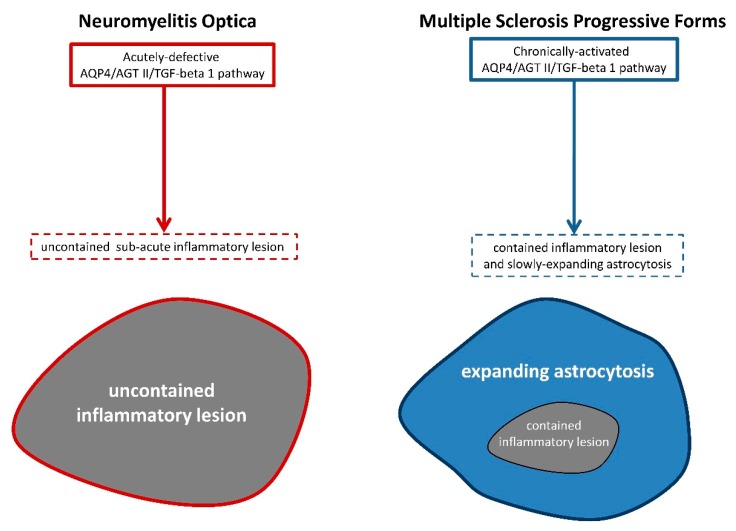
Due to the presence of autoantibodies directed against Aquaporin 4 (AQP4), the molecular pathway putatively linking AQP4, angiotensin II (AGT II) and the anti-inflammatory/progliotic molecule transforming growth factor beta 1 (TGF-beta 1) is ineffective in patients suffering from neuromyelitis optica. As a consequence, large uncontained inflammatory lesions develop in a sub-acute fashion. On the contrary, due to the overexpression of AQP4 on astrocytes localized in periplaque areas, plaque-associated inflammation remains contained by TGF-beta 1, however astrocytosis extends distance away from plaque borders.
